# Evaluating the role of BMI in survival and complications in older esophageal squamous cell carcinoma following esophagectomy

**DOI:** 10.3389/or.2026.1757530

**Published:** 2026-02-23

**Authors:** Kexun Li, Simiao Lu, Changding Li, Jie Mao, Huan Zhang, Kangning Wang, Guangyuan Liu, Yongtao Han, Lin Peng, Xuefeng Leng

**Affiliations:** 1 Department of Thoracic Surgery, Sichuan Clinical Research Center for Cancer, Sichuan Cancer Hospital and Institute, Sichuan Cancer Center, Affiliated Cancer Hospital of University of Electronic Science and Technology of China (Sichuan Cancer Hospital), Chengdu, China; 2 Department of Thoracic Surgery I, Third Affiliated Hospital of Kunming Medical University (Yunnan Cancer Hospital, Yunnan Cancer Center), Kunming, China; 3 School of Public Health, Chongqing Medical University, Chongqing, China; 4 Department of Thoracic Surgery, Zigong First People’s Hospital, Zigong, Sichuan, China

**Keywords:** body mass index, complication, elderly patients, esophageal squamous cell carcinoma, esophagectomy

## Abstract

**Background:**

To evaluate the impact of Body Mass Index (BMI) on survival and postoperative complications in older patients with esophageal squamous cell carcinoma (ESCC) following esophagectomy, we designed this study.

**Materials and methods:**

We retrospectively analyzed 469 patients aged ≥70 years with thoracic ESCC who underwent esophagectomy at Sichuan Cancer Hospital (May 2016–August 2021). Patients were grouped by WHO BMI categories: underweight (<18.5 kg/m^2^), normal (18.5–24.9 kg/m^2^), and overweight/obese (≥25 kg/m^2^). Primary outcomes were overall survival (OS) and disease-free survival (DFS); secondary outcomes included Clavien-Dindo grade III–IV complications. Kaplan-Meier, Cox models, and restricted cubic splines (RCS) were used.

**Results:**

Median follow-up was 47.5 months; R0 resection was achieved in 96.4%. BMI distribution: 7.3% low, 76.8% normal, 16.0% high. Median OS was 44.9 months overall, with no significant OS or DFS differences among BMI groups. RCS demonstrated a significant U-shaped association between continuous BMI and survival: protective ranges were approximately 21.9–27.0 kg/m^2^ for OS (P non-linearity = 0.014) and 20.2–27.2 kg/m^2^ for DFS (P non-linearity = 0.033).

**Conclusion:**

In elderly ESCC patients after esophagectomy, BMI does not independently influence OS or DFS, though low BMI is associated with specific serious complications. Perioperative optimization—particularly nutritional support for underweight patients—remains essential.

## 1 Introduction

Esophageal squamous cell carcinoma (ESCC) is one of the most prevalent and aggressive cancers in the elderly population, significantly affecting both survival rates and quality of life (1–3). As the global population ages, there is an increasing trend of older patients being diagnosed with esophageal cancer, which makes the management of these patients an area of growing concern (4–6). Surgical resection, particularly esophagectomy, remains a cornerstone in the treatment of localized ESCC, with the potential for curative outcomes. However, this complex surgical procedure is associated with high perioperative risks and can have a considerable impact on postoperative recovery, particularly in elderly patients (7–9). The treatment approach for ESCC typically combines surgery with neoadjuvant therapy, such as chemotherapy or chemoradiotherapy, followed by adjuvant treatments based on pathological results and the individual patient’s condition (10–12). This multimodal approach is consistent with the recommendations in current clinical guidelines, including those from the National Comprehensive Cancer Network (NCCN) and the Chinese Anti-Cancer Association (CACA) (13–15).

Body Mass Index (BMI), a widely recognized indicator of nutritional status and general health, has been studied in the context of various cancers, due to its potential influence on surgical outcomes, complications, and long-term survival (16–18). Nutritional status plays a crucial role in both survival and prognosis, with adequate nutrition being necessary for recovery and the management of cancer. BMI is one of the objective indicators that reflects the nutritional status of patients. While obesity is generally considered a risk factor for multiple comorbidities, including cardiovascular and metabolic diseases, low BMI and malnutrition are also associated with poor prognosis in cancer patients. Given the heterogeneity within the elderly population, it is essential to determine whether BMI serves as an independent prognostic factor, or if other factors such as frailty, comorbid conditions, and treatment strategies play a more substantial role in influencing outcomes.

This study seeks to address the knowledge gap regarding the impact of BMI on the postoperative outcomes of elderly patients with ESCC following esophagectomy. Through this analysis, we hope to provide critical insights that can guide clinical decision-making for elderly ESCC patients undergoing esophagectomy.

## 2 Materials and methods

### 2.1 Study design

**FIGURE 1:**
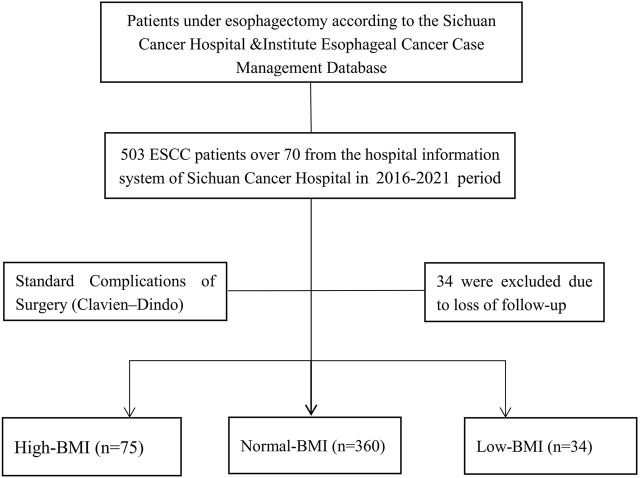
CONSORT diagram showing patient selection. ESCC, thoracic esophageal squamous cell carcinoma.

This retrospective cohort study utilized data from the Sichuan Cancer Hospital & Institute Esophageal Cancer Case Management Database (SCCH-ECCM Database). We included elderly patients, aged 70 years and older, diagnosed with thoracic ESCC who underwent esophagectomy from May 2016 to August 2021. Inclusion criteria required patients to have histologically confirmed ESCC with no evidence of distant metastasis as determined by clinical imaging techniques such as CT and ultrasound. Patients were excluded if they presented with non-thoracic esophageal tumors, other histological cancer types, incomplete clinical data, or lacked follow-up information (Figure 1). The final follow-up was concluded on 20 December 2023. Patients were categorized into groups based on their BMI: underweight (<18.5 kg/m^2^), normal weight (18.5–24.9 kg/m^2^), and overweight/obese (≥25 kg/m^2^) according to WHO guidelines (19,20). The primary outcomes were overall survival (OS) and disease-free survival (DFS), with secondary outcomes including postoperative complication rates. OS was defined from the date of surgery to death or last follow-up, while DFS was from surgery to recurrence, death, or last follow-up. Tumor staging followed the eighth edition UICC/AJCC TNM classification, and two independent pathologists validated pathological diagnoses (13).

### 2.2 Criteria and characteristics of adverse events

Postoperative complications were assessed using the Clavien-Dindo classification, where grades III and IV indicated severe complications necessitating invasive procedures or intensive care unit management (21–23). Perioperative mortality was defined as death occurring during hospitalization or within 30 days post-surgery. The study adhered to the STROBE guidelines for reporting observational studies (24).

### 2.3 Statistical analysis

Baseline characteristics among the BMI groups were compared using chi-square tests for categorical variables. Kaplan-Meier survival analyses were utilized to examine OS and DFS, with differences assessed through log-rank tests. Cox proportional hazards models identified independent prognostic factors for OS and DFS, adjusting for confounders such as age, sex, tumor stage, BMI, and comorbidities. A p-value of less than 0.05 was considered significant. Statistical analyses were conducted using SPSS version 26.0 and RStudio version 4.3.0.

### 2.4 Ethical considerations

The study received ethical approval from the Ethics Committee for Medical Research and New Medical Technology at Sichuan Cancer Hospital (Approval No. SCCHEC-02-2024-191). As a retrospective analysis, patient consent was waived, but strict confidentiality and data protection measures were implemented, complying with the Declaration of Helsinki (2013 revision). The authors ensured adherence to stringent methodological standards to uphold the validity and reliability of the study findings.

## 3 Results

### 3.1 Patient characteristics

A total of 469 elderly patients (aged ≥70 years) diagnosed with thoracic ESCC who underwent esophagectomy were included in the final analysis. Among these, 27 patients (5.8%) were aged over 80 years. R0 resection, indicating negative surgical margins, was achieved in 452 patients (96.4%), reflecting a high rate of complete tumor removal. Preoperative neoadjuvant therapy was administered to 72 patients (15.4%). Postoperative complications classified as Clavien-Dindo grade III-IV were observed in 233 patients (49.7%) (Table 1). In terms of perioperative mortality, 2 patients (0.4%) died during the immediate hospitalization period, 3 patients (0.6%) died within 30 days post-surgery, and 14 patients (3.0%) died within 90 days (Table 1). Based on BMI categorization, 34 patients (7.3%) were in the low-BMI group (<18.5 kg/m^2^), 360 patients (76.8%) were in the normal-BMI group (18.5–24.9 kg/m^2^), and 75 patients (16.0%) were classified as high-BMI (≥25 kg/m^2^) (Figure 1).

**TABLE 1:** Demographic characteristics of patients.

Characteristic	Total	Low-BMI (n = 34)	Normal-BMI (n = 360)	High-BMI (n = 75)
Sex				
Male	375	28 (82.35%)	294 (81.67%)	53 (70.67%)
Female	94	6 (17.65%)	66 (18.33%)	22 (29.33%)
Age, years				
Median (range)	73 (70–88)	73 (70–82)	73 (70–88)	73 (70–83)
<80	442	31 (91.18%)	339 (94.17%)	72 (96.00%)
≥80	27	3 (8.82%)	21 (5.83%)	3 (4.00%)
Smoking				
Yes	241	17 (50.00%)	191 (53.06%)	33 (44.00%)
No	228	17 (50.00%)	169 (46.94%)	42 (56.00%)
Alcohol				
Yes	245	17 (50.00%)	194 (53.89%)	34 (45.33%)
No	224	17 (50.00%)	166 (46.11%)	41 (54.67%)
Tumor location				
Upper	61	2 (5.88%)	47 (13.06%)	12 (16.00%)
Middle	207	18 (52.94%)	158 (43.89%)	31 (41.33%)
Lower	201	14 (41.18%)	155 (43.06%)	32 (42.67%)
KPS score				
≤80	119	10 (29.41%)	90 (25.00%)	19 (25.33%)
≥90	350	24 (70.59%)	270 (75.00%)	56 (74.67%)
Anastomosis location				
McKeown	407	30 (88.24%)	316 (87.78%)	61 (81.33%)
Lovr-Lewis	62	4 (11.76%)	44 (12.22%)	14 (18.67%)
Lymphovascular invasion				
Yes	164	13 (38.24%)	122 (33.89%)	29 (38.67%)
No	305	21 (61.76%)	238 (66.11%)	46 (61.33%)
Nerve invasion				
Yes	204	15 (44.12%)	157 (43.61%)	32 (42.67%)
No	265	19 (55.88%)	203 (56.39%)	43 (57.33%)
Complete resection				
R0	452	34 (100.00%)	344 (95.56%)	74 (98.67%)
R1/R2	17	0 (0.00%)	16 (4.44%)	1 (1.33%)
cT stage				
T1	29	2 (5.88%)	24 (6.67%)	3 (4.00%)
T2	67	8 (23.53%)	51 (14.17%)	8 (10.67%)
T3	324	21 (61.76%)	244 (67.78%)	59 (78.67%)
T4	49	3 (8.82%)	41 (11.39%)	5 (6.67%)
cN stage				
N0	81	8 (23.53%)	63 (17.50%)	10 (13.33%)
N1	282	22 (64.71%)	217 (60.28%)	43 (57.33%)
N2	100	4 (11.76%)	75 (20.83%)	21 (28.00%)
N3	6	0 (0.00%)	5 (1.39%)	1 (1.33%)
c8th TNM stage				
I	27	2 (5.88%)	22 (6.11%)	3 (4.00%)
II	100	10 (29.41%)	79 (21.94%)	11 (14.67%)
III	286	19 (55.88%)	212 (58.89%)	55 (73.33%)
IV	56	3 (8.82%)	47 (13.06%)	6 (8.00%)
Pathological differentiation grade				
Moderate or well G1-2	328	26 (76.47%)	248 (68.89%)	54 (72.00%)
Poor or undifferentiated G3-4	141	8 (23.53%)	112 (31.11%)	21 (28.00%)
Neoadjuvant therapy				
Yes	72	1 (2.94%)	55 (15.28%)	16 (21.33%)
No	397	33 (97.06%)	305 (84.72%)	59 (78.67%)
pT stage				
T0	12	0 (0.00%)	10 (2.78%)	2 (2.67%)
T1	73	6 (17.65%)	57 (15.83%)	10 (13.33%)
T2	94	7 (20.59%)	71 (19.72%)	16 (21.33%)
T3	269	20 (58.82%)	204 (56.67%)	45 (60.00%)
T4	21	1 (2.94%)	18 (5.00%)	2 (2.67%)
pN stage				
N0	244	16 (47.06%)	191 (53.06%)	37 (49.33%)
N1	135	10 (29.41%)	100 (27.78%)	25 (33.33%)
N2	68	7 (20.59%)	52 (14.44%)	9 (12.00%)
N3	22	1 (2.94%)	17 (4.72%)	4 (5.33%)
p8th TNM stage				
I	88	6 (17.65%)	68 (18.89%)	14 (18.67%)
II	154	11 (32.35%)	122 (33.89%)	21 (28.00%)
III	193	15 (44.12%)	144 (40.00%)	34 (45.33%)
IV	34	2 (5.88%)	26 (7.22%)	6 (8.00%)
Died in 30 days	3	0 (0.00%)	3 (0.83%)	0 (0.00%)
Died in 90 days	14	0 (0.00%)	14 (3.89%)	0 (0.00%)
Adverse events (Clavien–Dindo)				
0-II	234	16 (47.06%)	177 (49.17%)	41 (54.67%)
III-IV	233	18 (52.94%)	181 (50.28%)	34 (45.33%)
V	2	0 (0.00%)	2 (0.56%)	0 (0.00%)

### 3.2 Survival outcomes

**FIGURE 2:**
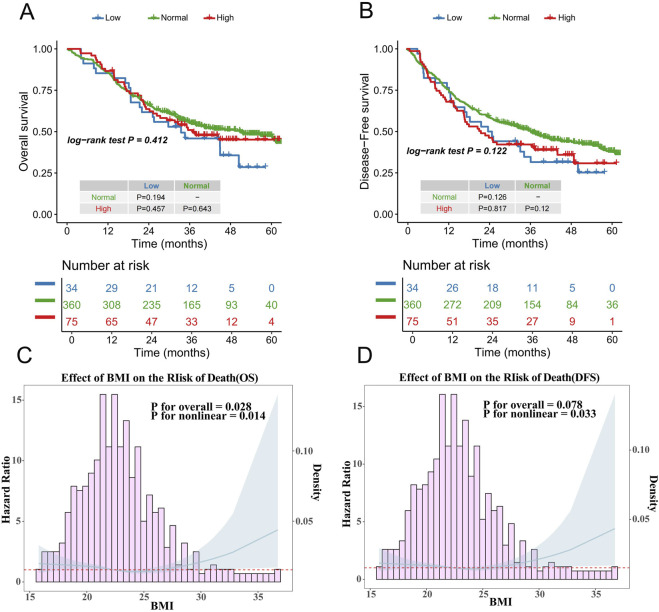
**(A)** Overall survival curve of different BMI groups; **(B)** Disease-free survival curve of different BMI groups; **(C)** Restricted cubic spline analysis for all patients between the BMI and the hazard ratios for OS; **(D)** Restricted cubic spline analysis for all patients between the BMI and the hazard ratios for DFS.

The median follow-up period for the 469 patients was 47.5 months, with a median OS time of 44.9 months. For patients with a normal BMI, the median OS was 50.7 months, with a 1-year OS rate of 86%, a 3-year OS rate of 55%, and a 5-year OS rate of 47%. Patients with a high BMI had a median OS of 36.9 months, a 1-year OS rate of 87%, a 3-year OS rate of 51%, and a 5-year OS rate of 45%. Those with a low BMI had a median OS of 33.5 months, with a 1-year OS rate of 85%, a 3-year OS rate of 46%, and a 5-year OS rate of 24%. There were no statistically significant differences in OS among the three BMI groups, as indicated by the following p-values: Normal-BMI versus High-BMI (P = 0.643), Normal-BMI versus Low-BMI (P = 0.194), and High-BMI versus Low-BMI (P = 0.457) (Figure 2A).

Regarding DFS, patients with a normal BMI had a median DFS of 36.5 months, with a 1-year DFS rate of 76%, a 3-year DFS rate of 51%, and a 5-year DFS rate of 38%. Those with a high BMI had a median DFS of 21.9 months, with a 1-year DFS rate of 68%, a 3-year DFS rate of 42%, and a 5-year DFS rate of 29%. Patients with a low BMI had a median DFS of 24.0 months, with a 1-year DFS rate of 76%, a 3-year DFS rate of 35%, and a 5-year DFS rate of 20%. Similar to OS, there were no statistically significant differences in DFS among the three groups: Normal-BMI versus High-BMI (P = 0.120), Normal-BMI versus Low-BMI (P = 0.126), and High-BMI versus Low-BMI (P = 0.817) (Figure 2B).

The median BMI of the cohort was 22.42 kg/m2 (Interquartile Range: 20.57–24.56). Restricted cubic spline analysis revealed a significant non-linear relationship between BMI and survival outcomes. For OS, a U-shaped curve was observed after adjustment (P for non-linearity = 0.014), with the HR found in patients with a BMI between 21.88 kg/m2 and 26.98 kg/m2 (HR <1). Similarly, DFS exhibited a comparable U-shaped pattern after adjustment (P for non-linearity = 0.033), demonstrating protective effects (HR <1) within the BMI range of 20.18 kg/m2 to 27.18 kg/m2 (Figures 2C,D).

### 3.3 Short-term outcomes and adverse events (Clavien-Dindo, 2009)

**FIGURE 3:**
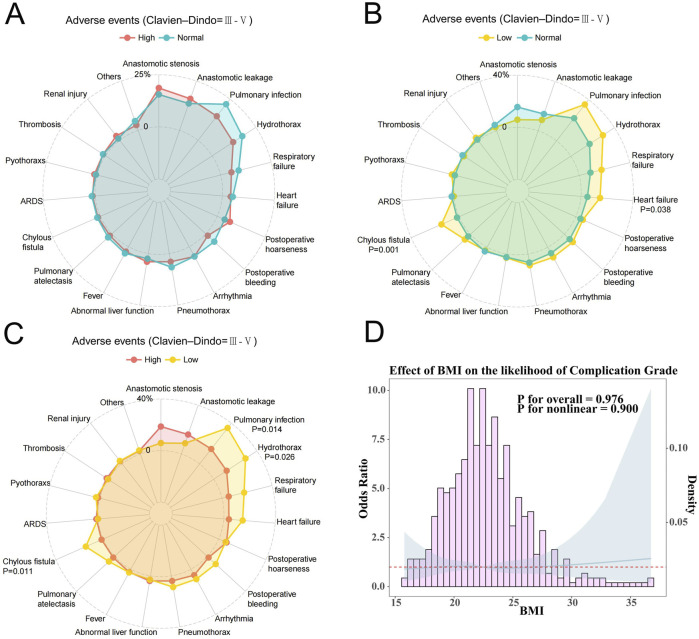
**(A)** Clavien-Dindo grade III-V complications of Normal and High BMI groups; **(B)** Clavien-Dindo grade III-V complications of Normal and High Low groups; **(C)** Clavien-Dindo grade III-V complications of Low and High BMI groups; **(D)** Restricted cubic spline analysis for all patients between the BMI and the odds ratios for complications.

In analyzing the short-term outcomes and adverse events, particularly those classified as Clavien-Dindo grade III and above, we found notable patterns among the different BMI groups. Importantly, there were no statistically significant differences in 30-day or 90-day mortality rates among patients with normal BMI, high BMI, and low BMI. Postoperative complications classified as Clavien-Dindo grades III-IV were observed in 18 patients (52.94%) with low BMI, 181 patients (50.28%) with normal BMI, and 34 patients (45.33%) with high BMI (Tables 1,3). For patients with low BMI, the incidence of certain complications was notably higher compared to those with normal and high BMI. Specifically, low BMI patients experienced elevated rates of pulmonary infections, hydrothorax, respiratory failure, heart failure, and chylous fistula. Compared to patients with normal BMI, the occurrence of heart failure (P = 0.038) and chylous fistula (P = 0.001) was significantly higher in the low BMI group. Furthermore, when compared with the high BMI group, low BMI patients had significantly higher incidences of pulmonary infection (P = 0.014), hydrothorax (P = 0.026), and chylous fistula (P = 0.011) (Figures 3A–C). Restricted cubic spline analysis further elucidated the relationship between BMI and the OR for postoperative complications. Although a non-linear trend was observed, the association did not reach statistical significance (P for non-linearity = 0.900) (Figure 3D). The curve demonstrated a relatively flat trajectory across the BMI spectrum, with the OR for complications remaining close to one in most ranges.

**TABLE 2:** Adverse events (Clavien–Dindo ≥III, 2009) of different BMI groups.

Adverse events	Low (n = 34)			Normal (n = 360)			High (n = 75)		
	III	IV	V	III	IV	V	III	IV	V
Anastomotic stenosis	2 (5.88%)	​	​	55 (15.28%)	1 (0.28%)	​	14 (18.67%)	​	​
Anastomotic leakage	3 (8.82%)	​	​	31 (8.61%)	17 (4.72%)	1 (0.28%)	9 (12.00%)	3 (4.00%)	​
Pulmonary infection	5 (14.71%)	7 (20.59%)	​	42 (11.67%)	35 (9.72%)	2 (0.56%)	4 (5.33%)	7 (9.33%)	​
Hydrothorax	10 (29.41%)	​	​	62 (17.22%)	​	​	9 (12.00%)	​	​
Respiratory failure	​	6 (17.65%)	​	3 (0.83%)	27 (7.50%)	2 (0.56%)	​	4 (5.33%)	​
Heart failure	5 (14.71%)	​	​	10 (2.78%)	7 (1.94%)	1 (0.28%)	2 (2.67%)	1 (1.33%)	​
Postoperative hoarseness	2 (5.88%)	​	​	12 (3.33%)	2 (0.56%)	​	4 (5.33%)	1 (1.33%)	​
Postoperative bleeding	2 (5.88%)	1 (2.94%)	​	14 (3.89%)	6 (1.67%)	​	1 (1.33%)	​	​
Arrhythmia	3 (8.82%)	​	​	16 (4.44%)	3 (0.83%)	​	2 (2.67%)	2 (2.67%)	​
Pneumothorax	3 (8.82%)	​	​	24 (6.67%)	​	​	3 (4.00%)	​	​
Abnormal liver function	1 (2.94%)	​	​	9 (2.50%)	2 (0.56%)	​	3 (4.00%)	​	​
Fever	1 (2.94%)	​	​	13 (3.61%)	​	​	2 (2.67%)	​	​
Pulmonary atelectasis	2 (5.88%)	​	​	9 (2.50%)	​	​	1 (1.33%)	​	​
Suspected anastomotic leakage	​	​	​	3 (0.83%)	​	​	​	​	​
Chylous fistula	4 (11.76%)	1 (2.94%)	​	2 (0.56%)	4 (1.11%)	​	1 (1.33%)	​	​
ARDS	​	​	​	​	6 (1.67%)	​	1 (1.33%)	​	​
Pyothoraxs	1 (2.94%)	​	​	1 (0.28%)	1 (0.28%)	​	​	1 (1.33%)	​
Wound infection	​	​	​	3 (0.83%)	​	​	​	2 (2.67%)	​
Pulmonary embolism	​	​	​	1 (0.28%)	1 (0.28%)	1 (0.28%)	​	​	​
Delirium	​	​	​	1 (0.28%)	1 (0.28%)	​	​	​	​
Thrombosis	​	​	​	5 (1.39%)	​	​	1 (1.33%)	​	​
Ketosis	​	1 (2.94%)	​	​	​	​	​	​	​
Renal injury	1 (2.94%)	​	​	2 (0.56%)	2 (0.56%)	​	2 (2.67%)	​	​
Tracheal injury	​	​	​	​	2 (0.56%)	1 (0.28%)	​	​	​
Cerebral infarction	​	​	​	1 (0.28%)	​	​	​	​	​
Gastric perforation	​	​	​	​	1 (0.28%)	​	​	​	​
Diaphragmatic hernia	​	​	​	​	1 (0.28%)	​	​	​	​

**TABLE 3:** Demographic characteristics of patients in Normal, High and Low groups.

Characteristic	Low-BMI (n = 34)	High-BMI (n = 75)	*P* value	Low-BMI (n = 34)	Normal-BMI (n = 360)	*P* value
Sex	​	​	0.196	​	​	0.921
Male	28 (82.35%)	53 (70.67%)	​	28 (82.35%)	294 (81.67%)	​
Female	6 (17.65%)	22 (29.33%)	​	6 (17.65%)	66 (18.33%)	​
Age, years	​	​	0.373	​	​	0.450
median (range)	73 (70–82)	73 (70–83)	​	73 (70–82)	73 (70–88)	​
<80	31 (91.18%)	72 (96.00%)	​	31 (91.18%)	339 (94.17%)	​
≥80	3 (8.82%)	3 (4.00%)	​	3 (8.82%)	21 (5.83%)	​
Smoking	​	​	0.560	​	​	0.733
Yes	17 (50.00%)	33 (44.00%)	​	17 (50.00%)	191 (53.06%)	​
No	17 (50.00%)	42 (56.00%)	​	17 (50.00%)	169 (46.94%)	​
Alcohol	​	​	0.651	​	​	0.664
Yes	17 (50.00%)	34 (45.33%)	​	17 (50.00%)	194 (53.89%)	​
No	17 (50.00%)	41 (54.67%)	​	17 (50.00%)	166 (46.11%)	​
Tumor location	​	​	0.276	​	​	0.390
Upper	2 (5.88%)	12 (16.00%)	​	2 (5.88%)	47 (13.06%)	​
Middle	18 (52.94%)	31 (41.33%)	​	18 (52.94%)	158 (43.89%)	​
Lower	14 (41.18%)	32 (42.67%)	​	14 (41.18%)	155 (43.06%)	​
KPS score	​	​	0.655	​	​	0.572
≤80	10 (29.41%)	19 (25.33%)	​	10 (29.41%)	90 (25.00%)	​
≥90	24 (70.59%)	56 (74.67%)	​	24 (70.59%)	270 (75.00%)	​
Anastomosis location	​	​	0.369	​	​	1.000
McKeown	30 (88.24%)	61 (81.33%)	​	30 (88.24%)	316 (87.78%)	​
Lovr-Lewis	4 (11.76%)	14 (18.67%)	​	4 (11.76%)	44 (12.22%)	​
Lymphovascular invasion	​	​	0.966	​	​	0.610
Yes	13 (38.24%)	29 (38.67%)	​	13 (38.24%)	122 (33.89%)	​
No	21 (61.76%)	46 (61.33%)	​	21 (61.76%)	238 (66.11%)	​
Nerve invasion	​	​	0.887	​	​	0.955
Yes	15 (44.12%)	32 (42.67%)	​	15 (44.12%)	157 (43.61%)	​
No	19 (55.88%)	43 (57.33%)	​	19 (55.88%)	203 (56.39%)	​
Complete resection	​	​	1.000	​	​	0.380
R0	34 (100.00%)	74 (98.67%)	​	34 (100.00%)	344 (95.56%)	​
R1/R2	0 (0.00%)	1 (1.33%)	​	0 (0.00%)	16 (4.44%)	​
Clinical T stage	​	​	0.275	​	​	0.532
T1	2 (5.88%)	3 (4.00%)	​	2 (5.88%)	24 (6.67%)	​
T2	8 (23.53%)	8 (10.67%)	​	8 (23.53%)	51 (14.17%)	​
T3	21 (61.76%)	59 (78.67%)	​	21 (61.76%)	244 (67.78%)	​
T4	3 (8.82%)	5 (6.67%)	​	3 (8.82%)	41 (11.39%)	​
N stage	​	​	0.185	​	​	0.480
N0	8 (23.53%)	10 (13.33%)	​	8 (23.53%)	63 (17.50%)	​
N1	22 (64.71%)	43 (57.33%)	​	22 (64.71%)	217 (60.28%)	​
N2	4 (11.76%)	21 (28.00%)	​	4 (11.76%)	75 (20.83%)	​
N3	0 (0.00%)	1 (1.33%)	​	0 (0.00%)	5 (1.39%)	​
8th TNM stage	​	​	0.274	​	​	0.740
I	2 (5.88%)	3 (4.00%)	​	2 (5.88%)	22 (6.11%)	​
II	10 (29.41%)	11 (14.67%)	​	10 (29.41%)	79 (21.94%)	​
III	19 (55.88%)	55 (73.33%)	​	19 (55.88%)	212 (58.89%)	​
IV	3 (8.82%)	6 (8.00%)	​	3 (8.82%)	47 (13.06%)	​
Pathological differentiation grade	​	​	0.625	​	​	0.359
Moderate or well G1-2	26 (76.47%)	54 (72.00%)	​	26 (76.47%)	248 (68.89%)	​
Poor or undifferentiated G3-4	8 (23.53%)	21 (28.00%)	​	8 (23.53%)	112 (31.11%)	​
Neoadjuvant therapy	​	​	0.014	​	​	0.067
Yes	1 (2.94%)	16 (21.33%)	​	1 (2.94%)	55 (15.28%)	​
No	33 (97.06%)	59 (78.67%)	​	33 (97.06%)	305 (84.72%)	​
Pathological T stage	​	​	0.875	​	​	0.858
T0	0 (0.00%)	2 (2.67%)	​	0 (0.00%)	10 (2.78%)	​
T1	6 (17.65%)	10 (13.33%)	​	6 (17.65%)	57 (15.83%)	​
T2	7 (20.59%)	16 (21.33%)	​	7 (20.59%)	71 (19.72%)	​
T3	20 (58.82%)	45 (60.00%)	​	20 (58.82%)	204 (56.67%)	​
T4	1 (2.94%)	2 (2.67%)	​	1 (2.94%)	18 (5.00%)	​
Pathological N stage	​	​	0.658	​	​	0.743
N0	16 (47.06%)	37 (49.33%)	​	16 (47.06%)	191 (53.06%)	​
N1	10 (29.41%)	25 (33.33%)	​	10 (29.41%)	100 (27.78%)	​
N2	7 (20.59%)	9 (12.00%)	​	7 (20.59%)	52 (14.44%)	​
N3	1 (2.94%)	4 (5.33%)	​	1 (2.94%)	17 (4.72%)	​
Pathological 8th TNM stage	​	​	0.957	​	​	0.968
I	6 (17.65%)	14 (18.67%)	​	6 (17.65%)	68 (18.89%)	​
II	11 (32.35%)	21 (28.00%)	​	11 (32.35%)	122 (33.89%)	​
III	15 (44.12%)	34 (45.33%)	​	15 (44.12%)	144 (40.00%)	​
IV	2 (5.88%)	6 (8.00%)	​	2 (5.88%)	26 (7.22%)	​
Died in 30 days	0 (0.00%)	0 (0.00%)	1.000	0 (0.00%)	3 (0.83%)	1.000
Died in 90 days	0 (0.00%)	0 (0.00%)	1.000	0 (0.00%)	14 (3.89%)	0.621
Adverse events (Clavien–Dindo)	​	​	0.461	​	​	0.878
0-II	16 (47.06%)	41 (54.67%)	​	16 (47.06%)	177 (49.17%)	​
III-IV	18 (52.94%)	34 (45.33%)	​	18 (52.94%)	181 (50.28%)	​
V	0 (0.00%)	0 (0.00%)	​	0 (0.00%)	2 (0.56%)	​

### 3.4 Risk factors

**FIGURE 4:**
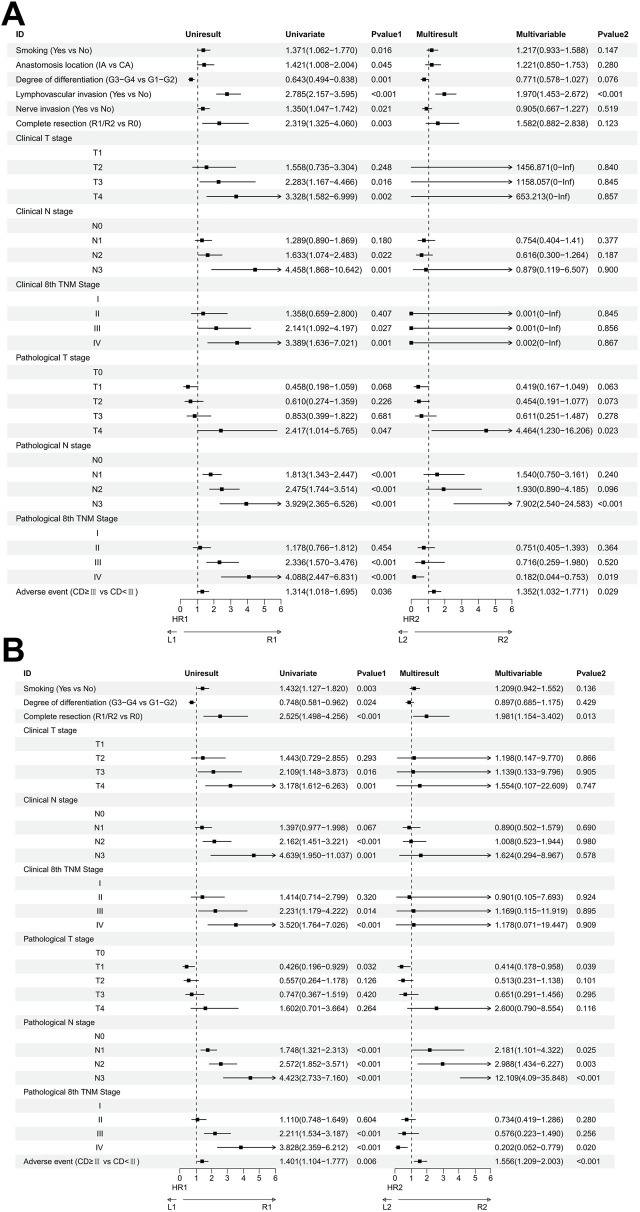
**(A)** Univariate and multivariate Cox regression analyses for factors affecting OS of patients; **(B)** Univariate and multivariate Cox regression analyses for factors affecting OS of patients.

Univariate analysis revealed several factors influencing OS. These included smoking status (Yes vs. No), anastomosis location (IA vs. CA), degree of differentiation (G3-G4 vs. G1-G2), lymphovascular invasion (Yes vs. No), nerve invasion (Yes vs. No), completeness of resection (R1/R2 vs. R0), occurrence of adverse events (Clavien-Dindo grade ≥III vs. <III), clinical stage, and pathological stage. Further multivariate analysis identified lymphovascular invasion (P < 0.001), pathological T4 stage (P = 0.023), pathological N3 stage (P < 0.001), and pathological IV stage (P = 0.019) as the most critical factors affecting OS (Figure 4A).

Similarly, for DFS, univariate analysis indicated that smoking, degree of differentiation, completeness of resection, adverse events, clinical stage, and pathological stage were influential. Multivariate analysis further highlighted that incomplete resection (R1/R2) (P = 0.013), pathological N2 stage (P = 0.003), pathological N3 stage (P < 0.001), pathological III stage (P = 0.020), and pathological IV stage (P < 0.001) were the most crucial factors impacting DFS (Figure 4B).

## 4 Discussion

**FIGURE 5:**
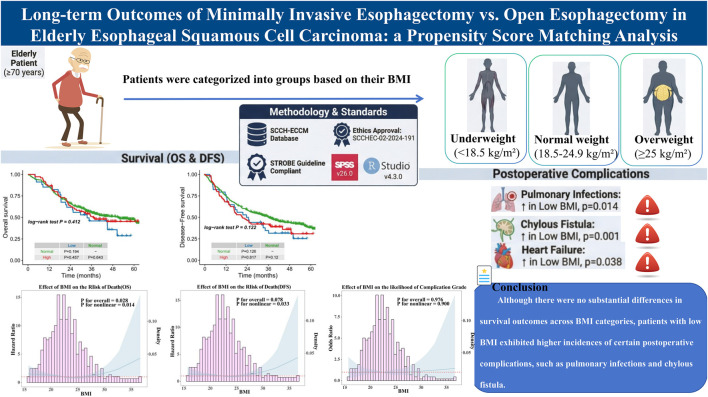
Visual abstract of impact of BMI on elderly patients after esophagectomy.

This study aimed to evaluate the impact of BMI on the postoperative outcomes of elderly patients with ESCC following esophagectomy. Outcomes showed no significant differences in OS and DFS among the BMI groups (normal, low, and high BMI). These findings suggest that while BMI is a recognized indicator of nutritional status and health, it may not independently predict long-term survival outcomes in elderly ESCC patients who undergo esophagectomy (Figure 5).

There were no significant differences in preoperative characteristics between patients with normal and low BMI, while patients with high and low BMI showed no significant differences in various indicators except for the administration of neoadjuvant therapy. Neoadjuvant therapy, as supported by findings from NEOCRTEC5010, has demonstrated significant advantages in extending survival time and reducing postoperative tumor recurrence without increasing postoperative complication rates or perioperative mortality (25). This advantage explains why our study did not perform further propensity score matching (PSM) to adjust for these differences in neoadjuvant treatment across BMI groups.

Interestingly, however, patients with a low BMI experienced significantly higher incidences of several specific postoperative complications, including pulmonary infections, hydrothorax, respiratory failure, heart failure, and chylous fistula compared to those with normal or high BMI. These findings highlight the heightened vulnerability of underweight patients to certain adverse events, possibly due to diminished physiological reserves and poorer nutritional status, which can impair recovery and increase susceptibility to complications (16–18).

In line with these findings, a study from Japan reveals a nuanced U-shaped association between BMI and outcomes such as mortality and major complications, highlighting that patients with both low and high BMI are at increased risk. Importantly, severely underweight individuals (BMI <16.0 kg/m^2^) exhibited a more than twofold increase in mortality risk, underscoring the vulnerability linked to low BMI. Conversely, obesity (BMI ≥27.5 kg/m^2^) also elevated mortality risk, though to a lesser extent. This indicates that extremes in BMI can contribute to poorer outcomes following esophagectomy in ESCC patients. Moreover, the study identified a reverse J-shaped relationship between BMI and failure to rescue, suggesting that lower BMI patients face greater challenges in recovery after major complications arise. This emphasizes the need for meticulous nutritional and postoperative care, especially for underweight patients, to improve resilience to postoperative complications. Ensuring adequate nutritional support and monitoring in the perioperative period could potentially mitigate the elevated risks associated with low BMI. For patients with high BMI, weight management strategies and careful monitoring for obesity-related complications could be beneficial (26).

In Mitzman et al.'s study, significant differences were observed across BMI categories for seven out of nine types of complications. Specifically, underweight and obesity class III patients were associated with an increased rate of complications. Conversely, individuals in the overweight and obesity class I categories tended to have a reduced risk for most types of complications. These insights align with our study’s results, reinforcing the notion that while BMI does not independently predict long-term survival outcomes in elderly ESCC patients undergoing esophagectomy, it does have a notable impact on the risk of postoperative complications. This highlights the complexity of BMI’s role, underscoring the importance of a comprehensive preoperative assessment that considers BMI as one of several factors influencing patient outcomes (27).

The prognostic significance of BMI in esophageal cancer remains controversial. Several previous reports have described an “obesity paradox,” in which overweight or mildly obese patients show improved survival compared to those with normal or low BMI, potentially due to greater nutritional reserves and better tolerance of surgical and adjuvant treatments. In contrast, other studies have reported that both low and high BMI are associated with worse survival, showing a U-shaped or reverse J-shaped relationship between BMI and mortality. Still, a number of studies—like ours—have found no clear survival differences between BMI categories. This variability across studies underscores the complexity of BMI as a prognostic factor in esophageal cancer. The relationship between BMI and prognosis in esophageal cancer should be interpreted in light of body composition rather than BMI alone. BMI does not distinguish between lean mass and fat mass, and low muscle mass (sarcopenia) has been independently linked to worse cancer outcomes. Future studies incorporating skeletal muscle measurements from CT imaging or bioelectrical impedance could help clarify whether muscle depletion, rather than low BMI *per se*, drives poor outcomes. Moreover, standardizing BMI cutoff values based on regional population characteristics may improve comparability across studies. Multicenter prospective research is warranted to disentangle the interplay of nutritional status, physiological reserve, and metabolic health in determining both perioperative safety and long-term prognosis.

The comparable distribution of stage III-IV disease—and the lack of a significantly higher proportion of stage IV cancer in the low-BMI group—strongly suggests that pretreatment low BMI in our elderly ESCC cohort did not predominantly stem from significantly more advanced tumor burden. This is noteworthy given our routine practice of preoperative nutritional optimization for patients with low BMI or poor nutritional status, which likely mitigated the impact of any existing tumor-related obstruction on nutritional parameters at the time of surgery. Therefore, the observed significantly higher rates of specific postoperative complications (pulmonary infections, hydrothorax, respiratory failure, heart failure, chylous fistula) in the low-BMI group appear less likely to be primarily driven by differences in tumor stage at the time of surgery. Instead, these complications seem more directly attributable to the physiological vulnerability associated with a low BMI itself, such as diminished physiological reserves and poorer baseline nutritional status, as previously hypothesized [16-18]. This aligns with the concept highlighted in the Japanese study [26] regarding the heightened challenges in recovery (failure to rescue) faced by underweight patients. While our nutritional interventions prior to surgery were essential, they may not have fully reversed the underlying frailty associated with chronic low BMI in the short preoperative window. In our cohort, we observed a notable imbalance in BMI distribution, with the majority of patients (approximately 75%) classified as having a normal BMI. This raises the question: Is it appropriate to directly apply the WHO BMI classification—developed primarily for Western populations—to East Asian populations without modification? While the suitability of WHO criteria for Asian populations, particularly Chinese individuals, remains debated, no universally accepted, unified BMI classification standard specific to Asian/Chinese populations currently exists. Importantly, Chinese clinical and nutritional guidelines, such as those issued by the Chinese Nutrition Society, continue to adopt the WHO BMI criteria [28]. To maintain consistency with domestic guideline-based practice and facilitate comparability with existing literature, we adhered to the WHO classification in this study. However, we acknowledge the objective limitations of applying categorical cutoffs that may not fully capture the nuances of BMI-outcome relationships in East Asian populations. To address this concern and reduce potential misclassification bias, we supplemented our categorical analysis with restricted cubic spline (RCS) analyses. These analyses allowed us to model BMI as a continuous variable, providing a more detailed characterization of its association with OS, DFS, and postoperative complications across its entire range (Figures 2C, 2D, and 3D). The RCS curves offer a visual and statistical interpretation beyond predefined categories, helping to elucidate potential non-linear relationships and mitigate concerns about the applicability of WHO cutoffs.

This study have several limitations. Firstly, as a retrospective cohort study, it is subject to inherent biases related to data collection and patient selection, which may affect the generalizability of the findings. Additionally, the study relies on data from a single institution, which may not be representative of wider populations or different healthcare settings. Another limitation is the reliance on BMI as the sole indicator of nutritional status, which does not account for other factors such as muscle mass or distribution of body fat, potentially limiting the comprehensiveness of assessing patients’ nutritional and health status. Furthermore, the study does not account for potential confounding factors such as socioeconomic status or lifestyle factors that could influence both BMI and cancer outcomes. Further research is warranted to explore these complex interactions and inform more personalized treatment approaches.

## 5 Conclusions

In conclusion, outcomes suggested that BMI, despite being a key indicator of nutritional status, does not significantly influence OS or DFS in this patient population. Although there were no substantial differences in survival outcomes across BMI categories, patients with low BMI exhibited higher incidences of certain postoperative complications, such as pulmonary infections and chylous fistula.

## Data Availability

The raw data supporting the conclusions of this article will be made available by the authors, without undue reservation.
